# An Analysis of Cost-Effectiveness of Stents Used in the Treatment of Coronary Artery Disease

**DOI:** 10.4274/balkanmedj.galenos.2019.2018.12.28

**Published:** 2019-08-22

**Authors:** Ferda Işıkçelik, İsmail Ağırbaş, Cansın Tulunay Kaya

**Affiliations:** 1 Department of Health Management, Ankara University School of Health Science, Ankara, Turkey; 2 Department of Cardiology, Ankara University School of Medicine, Ankara, Turkey

**Keywords:** Coronary artery disease, cost-effectiveness analysis, economic evaluation, health technology assessment, stents

## Abstract

**Background:**

There is only limited information about the cost-effectiveness of drug-eluting stents compared with bare-metal stents in Turkey.

**Aims:**

To evaluate bare-metal and drug-eluting stents used in the treatment of coronary artery disease from the perspective of the reimbursement institution with cost-effectiveness analysis.

**Study Design:**

Retrospective cost-effectiveness analysis.

**Methods:**

In our study, 329 patients diagnosed with coronary artery disease and treated with bare-metal or drug-eluting stents in the cardiology clinics of a public university hospital between January 1 and December 31, 2016 were investigated. Bare-metal and drug-eluting stents used in the treatment of coronary artery disease were evaluated retrospectively with cost-effectiveness analysis from the perspective of the reimbursement institution.

**Results:**

The cost of treatment with a bare-metal stent was 2,131.41 Turkish Liras, and the cost of treatment with a drug-eluting stent was 3,546.14 Turkish Liras; the Quality Adjusted Life Years value of treatment with a bare-metal stent was 0.8371, and the Quality Adjusted Life Years value of treatment with a drug-eluting stent was 0.8924. All these data were analyzed by decision tree. As a result of decision tree analysis, the weighted cost of treatment with a bare-metal stent was 2,340.71 Turkish Liras and weighted Quality Adjusted Life Years value was 0.8332; and the weighted cost of treatment with drug-eluting stent was 3,970.90 Turkish Liras and the weighted Quality Adjusted Life Years value of the treatment with drug-eluting stent was 0.8911. With these values, the additional cost-effectiveness ratio was calculated as 28,179.12 Turkish Liras per acquired Quality Adjusted Life Years. The additional cost-effectiveness ratio is in the first zone in the cost-effectiveness plane and below the very threshold of cost-effectiveness.

**Conclusion:**

In our study, it was concluded that drug-eluting stents are cost effective compared with bare-metal stents in the treatment of coronary artery disease. Considering the cost and effectiveness of the drug-eluting stent, it is thought that increasing reimbursement for this technology by the reimbursement agency would be beneficial for the service provider.

With the increasing average life expectancy and improved treatment methods, the number of elderly people who are prone to recurrent diseases has also increased (1). With the development of technology, a health service has become available with many technologies or interventions. Novel technologies are considered as one of the causes of rapidly increasing health expenditures (2). Scarce resources and increased health expenditures in the provision of health services are a well known fact. The health economy (3), which deals with the distribution and allocation of health services in a society, has attempted to provide a solution to this problem through health technology assessment. With health technology assessment studies, distinct alternatives in delivering a health service can be compared in terms of cost and effectiveness.

According to the Global Burden of Disease Study (4), cardiovascular disease was the worldwide leading cause of mortality among the top five diseases leading to the highest loss of life in 2016 (5). In National Burden of Disease studies carried out in Turkey, cardiovascular diseases were seen to take the first place among the 25 DALY causes in 2013 as well as in 2000 (6). Coronary artery disease (CAD) is one of the important causes of cardiovascular mortality worldwide (1,7,8,9). Considering the resources used for diagnosis and treatment of this disease, which has been becoming widespread in developed and developing countries (9), it is observed that the cost of this disease has a high share in health systems.

In order to reduce the recurrent revascularization process in CAD treatment, the drug-eluting stent (DES) has been developed as an alternative to a bare-metal stent (BMS). The use of DES in the treatment of CAD shows more effective results than BMS in terms of restenosis and recurrent revascularization (10,11). Because DES is more costly than BMS, some restrictions and arrangements have been made by the Social Security Institution with regard to reimbursement. In order to use DES in CAD treatment, the conditions specified in the National Communiqué on Healthcare Implementation must be met. With the latest amendment made in the National Communiqué on Healthcare Implementation and published in the Official Gazette No. 30175, dated 09/09/2017 (12), the material repayment amount for KR1151 coded DES was reduced from 1,018.00 Turkish Liras (TL) to 650.00 TL.

Our study was carried out with the aim of comparing BMS and DES, which are used in CAD treatment by using cost-effectiveness analysis from the perspective of Social Security Institution, the reimbursement institution, evaluating the burden of disease on the national budget in case of treatment with these technologies and calculating the effects of related technologies on quality of life. Our study is original in this respect.

## MATERIALS AND METHODS

In our study, BMSs and DESs, which are used in the treatment of CAD, were evaluated retrospectively with cost-effectiveness analysis from the Social Security Institution perspective. The study population was composed of 329 people with a diagnosis of CAD in cardiology clinics of university hospitals between January 1 and December 31, 2016. BMS was used in 102 patients and DES in 227 patients. The sampling procedure was not done in the study and the whole universe was included in the study. In the scope of the study, telephone calls were made to implement the EuroQol 5D 5L Health Questionnaire. Some prospective subjects could not be reached due to incorrect or incomplete contact information. As a result of telephone calls, 130 patients (68.42%) treated with DES and 60 patients (31.58%) treated with BMS were reached. Ethical approval for the study (date: 19.04.2017, number: 85434274-050.04.04/27789) was obtained from the Ankara University Ethics Committee.

Patient billing data obtained from the hospital data management system were classified under the headings of anesthesia, surgery, framework agreement services, pharmacy, cardiology, blood bank, laboratory, radiology, medical equipment, bedding and other expenses, and total expenditures by means of Microsoft Excel 2016. The total expenditures were calculated separately for the two comparison groups. The share of these expenditure items in the total costs and the average expenditure per capita on each of these items were determined. The average cost per patient was calculated for both of these health technologies.

In the study, Quality Adjusted Life Years (QALY) was used as a measure of effectiveness. In this context, QALY calculations were performed for BMS and DES by administering the “EuroQol 5D 5L Health Questionnaire” to the patients in the study by telephone. The EuroQol 5D 5L Health Questionnaire consists of five dimensions: mobility, self-care, normal work, pain/discomfort, and anxiety/status of low spirit. These five dimensions are scored on a 5-point Likert scale. A 5-digit score is obtained when the responder gives a score for all dimensions. This score is assessed by country-specific weightings on a standard scale, where a measurement of “0” indicates death and “1” indicates excellent health. Quality of life weights for the EuroQol 5D 5L Health Questionnaire for Turkey are not available. Therefore, the evaluation was conducted with the weights of countries that are frequently used in cost-effectiveness studies and health structures most resembling that of Turkey. Germany’s quality of life weights were used for QALY calculation, and Dutch quality of life weights were used for sensitivity analysis. In addition, for the comparison of effectiveness, hospital admissions and mean hospitalization days were analyzed for the same health problem in the last year after the treatment period of the patients.

The costs, effectiveness, possible results, and realization possibilities of BMS and DES were determined, and two different decision trees based on cost and QALY values were formed. The decision tree was drawn with the Precision Tree 7.5 extension of the Microsoft Excel Program, and weighted cost and effectiveness values were obtained for use in calculating the incremental cost-effectiveness ratio. The weighted cost was calculated by multiplying the costs of the DES and BMS used in CAD treatment by the probability of occurrence. The weighted QALY values were calculated by multiplying the QALY values of DES and BMS by the probability of occurrence. After the Incremental Cost-Effectiveness Ratio (ICER) was calculated, this ratio was assessed by the threshold value to determine whether alternative technology was included within acceptable limits for cost-effectiveness. Gross Domestic Product per Capita was taken as a threshold value indicator according to World Health Organization recommendations (13). The threshold value calculated by the effective sales rate (moving average) of the Central Bank in 2016 was evaluated as the threshold of the very cost-effectiveness and the threshold of three times of the value effectiveness.

In our study, due to the use of data from a single year, no reduction process was performed. One-way sensitivity analysis was implemented to measure the sensitivity of the study results to possible uncertainties. In the case of the use of BMS or DES in the treatment of CAD, a budget effect analysis was conducted to determine the burden on the national budget. The incidence figures of CAD in Turkey and those reportd by Onat et al. (14) were used to analyze the budget effect. The effect on the country’s budget is calculated if patients are treated with these technologies.

## RESULTS

In the scope of the study, 60 people (31.58%) treated with DES and 130 people (68.42%) treated with BMS were reached. In all, 31.67% of patients treated with BMS were female and 68.33% were male, whereas 30% of patients treated with DES were female and 70% were male. When the age distribution was examined, 50% of the BMS group was under 65 years of age, 50% was 65 years of age and over, 56.15% of the DES group was under 65 years of age, and 43.85% were 65 years of age and over. When the treatment units were examined, 81.67% of the BMS group was hospitalized in the clinic and 18.33% was in the intensive care unit; however, 100% of the DES group were inpatients in the clinic. Of the patients in the BMS group, 58.33% had concomitant diseases and 41.67% did not. In the DES group, 62% of the patients had concomitant diseases and 38% did not (Table 1).

Looking at the cost findings in Table 2, BMS technology has a total cost of 127,884.51 TL and an average cost of 2,131.41 TL per patient. It was determined that the expense item occupying the largest share of the total cost was medical equipment, with a proportion of 49.44%. This was followed respectively by surgery with 12.66%, cardiology with 11.63%, pharmacy with 7.47%, hospital bed costs with 6.14%, laboratory investigations with 5.40%, framework agreement services with 4.34%, blood bank with 0.68%, anesthesia with 0.39%, radiologic investigations with 0.23%, and miscellaneous costs with 1.62% (pain treatment applications, nephrology, physical therapy, outpatient clinic). As for DES technology, it has a total cost of 460,997.75 TL and an average cost of 3,546.14 TL per patient. It was determined that the expense item with the largest share of total costs in this group was also medical supplies with a ratio of 70.25%. This was followed respectively by surgery with 12.29%, drug costs with 4.69%, framework agreement services with 3.75%, cardiology services with 3.19%, laboratory investigations with 2.76%, hospital bed costs with 2.15%, blood bank with 0.2%, anesthesia with 0.06%, radiologic investigations with 0.02%, and miscellenaous costs with 0.51%.

Table 3 shows the effectiveness findings of the study. The QALY value of treatment with BMS was calculated as 0.8371, and the QALY value of treatment with DES was calculated as 0.8924. Within the scope of the visual analog scale, which is the second part of the EuroQol 5D 5L Health Questionnaire, patients were asked to give their current health a score on a scale of zero (the worst health level imaginable) to 100 (the best health level imaginable). According to the patients’ responses, the mean visual analog scale value of patients treated with BMS was 64.03, and the mean visual analog scale value of patients treated with DES was 72.56. When hospital visits for the same health problem in the year after the treatment period were investigated, it was observed that 35.2% of BMS users and 19.23% of those who used DES were re-admitted. The mean duration of hospitalization of the BMS group was 1.93 days, while the mean hospitalization duration of the DES group was 1.59 days.

**Figure 1:**
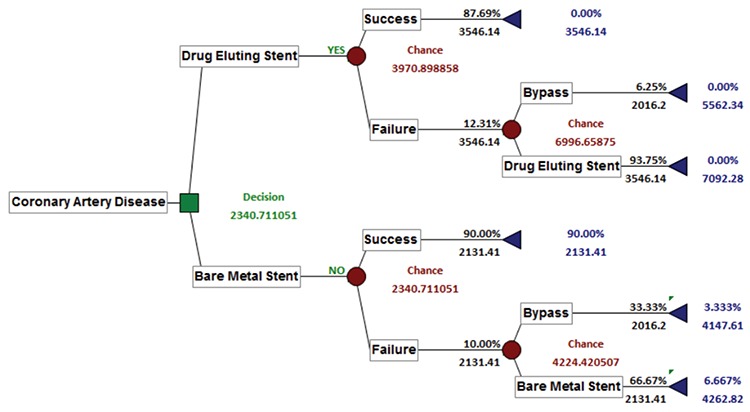
Cost decision tree.

Figure 1 shows the cost decision tree. Accordingly, the weighted cost value of BMS technology is 2,340.71 TL, and the weighted value of DES technology is 3,970.90 TL. As a requirement of cost-effectiveness analysis, the low-cost alternative was chosen, and the model determined the BMS as the decision.

**Figure 2:**
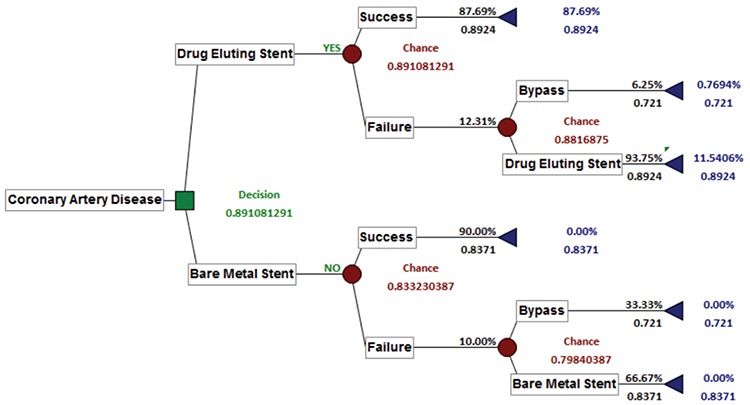
Effectiveness (Quality Adjusted Life Years) decision tree.

Figure 2 shows the effectiveness decision tree. Accordingly, it is seen that the BMS weighted QALY value is 0.8332 and the DES weighted QALY value is 0.8911. As a requirement of cost-effectiveness analysis, the alternative with high effectiveness was chosen; the model determined DES as the decision.

In order to determine the necessary additional cost to obtain additional QALY with DES technology compared with BMS technology, ICER was calculated from weighted cost and effectiveness values obtained by the decision tree. It was observed that DES required an incremental cost of 1,630.19 TL compared with BMS. When the effectivenss of the two technologies were compared, it was found that the QALY value of DES was 0.0579 units more than that of BMS. ICER for treatment with DES versus BMS technology for the management of CAD was 28,179.12 TL per acquired QALY. This means that treatment with DES technology requires an incremental cost of 28,179.12 TL to achieve additional QALY (Table 4).

**Figure 3:**
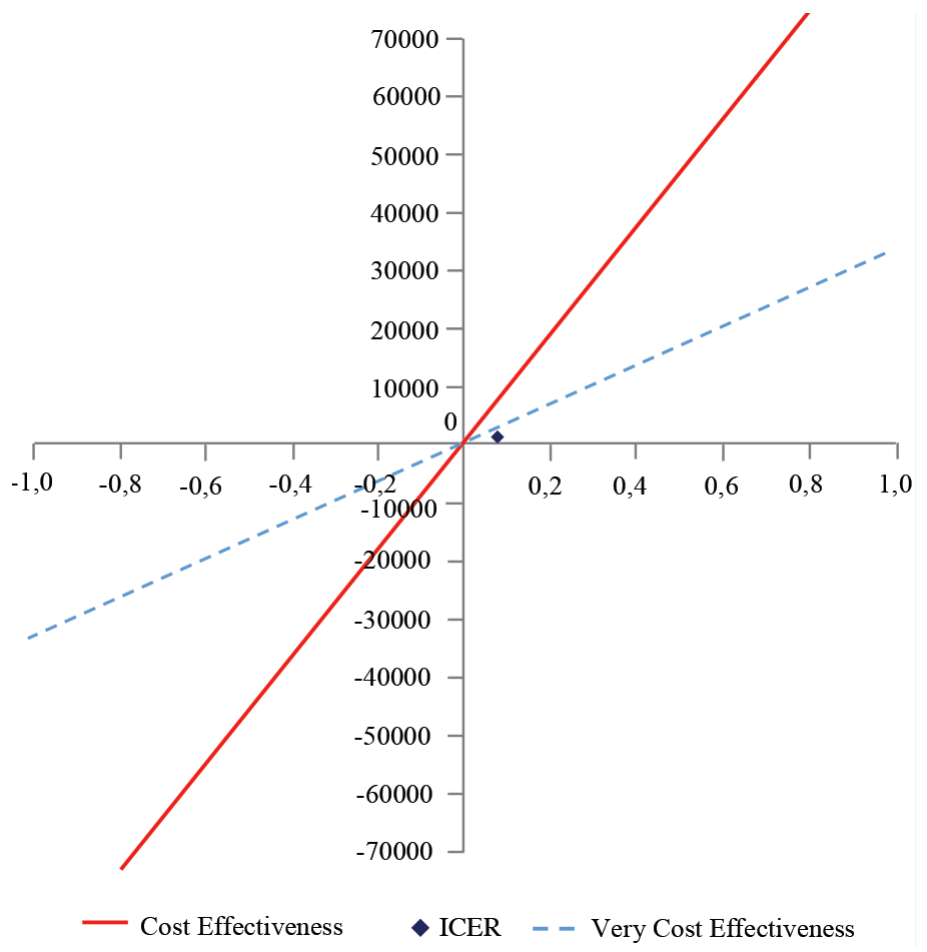
Cost effectiveness plane.

In order to determine whether a technology is cost effective, ICER is compared with the threshold. World Health Organization's recommends using GDP per capita as a threshold. If ICER is lower than GDP per capita, the cost effectiveness is very high, if it is between 1-3 times the GDP per capita, it is cost effective and if it is more than 3 times, it it not cost effective. According to Turkish Statistical Institute-TÜİK’s 2016 data, GDP per capita is 32,975.49 TL ($10,883). In this respect, the very threshold of cost-effectiveness was calculated as 32,975.49 TL and the cost-effectiveness threshold was calculated as 98,926.47 TL. With reference to this information, if ICER is less than TL 32,975.49, alternative technology is considered to be very cost effective, it is cost effective with an ICER of between 32,975.49 TL and 98,926.47 TL and not cost effective if the ICER is more than 98,926.47 TL. In the cost-effectiveness plane in Figure 3, ICER ranks in the first zone and below the very cost-effectiveness threshold. This situation implies that DES is more costly and more effective than BMS. Based on the position taken by ICER in the cost-effectiveness plane, DES was found to be very cost effective compared with BMS.

One-way sensitivity analysis was performed to measure the sensitivity of the results of cost-effectiveness analysis to unpredictable uncertainties. In this context, the cost and effectiveness findings of the study were changed. When the cost of treatment with DES was increased by 10%, the additional cost was 2,025.72 TL, and the ICER was 35,016.26 TL per QALY. When this was increased by 20%, the additional cost was 2,421.27 TL, and the ICER is 41,853.58 TL per QALY. When Dutch quality of life weights are used instead of Germany, the additional effectiveness ratio was calculated as 0.1169 and ICER was calculated as 13,940.31 TL per QALY earned. As a result, ICER was found to vary between 13,940.31 TL and 41,853.58 TL. When the cost of DES was increased by 10% in the one-way sensitivity analysis, and the QALY was calculated using Dutch weights, ICER remained very cost effective. With a 20% increase in DES cost, ICER approached the cost-effectiveness threshold from the very cost-effectiveness threshold (Table 5).

A budget effect analysis was performed as a complement to the cost-effectiveness assessment of the DES and BMS used in CAD treatment, and the burden of CAD on the country budget was calculated when CAD was treated with these health technologies. The incidence of CAD in Turkey is 2.5% (14). Turkey’s population is described by TÜİK as 79,814,871 people, as of December 31, 2016. To estimate the number of patients with coronary heart disease, the incidence of CAD is scaled to Turkey’s population and is calculated to be 1,995,372 people. Table 6 presents the results of the budget impact analysis from the Social Security Institution perspective, in order to evaluate the burden on the country’s budget in the case of the use of BMSs and medicated stents in the treatment of CAD. It is seen that CAD has a share of 3.90% of total health expenditures in the case of treatment with BMS and a share of 6.62% of total health expenditures where treated with DES is applied.

## DISCUSSION

In our study, we aimed to compare the cost and effectiveness of BMSs and DESs used in the treatment of CAD, to determine whether DES is cost effective compared with BMS and to determine the burden of using these health technologies in treatment, on the country’s budget. When the costs of these two health technologies are compared, it is concluded that DES is more costly than BMS. In a survey of the related literature, the cost of DES was found to be greater than that of BMS in studies with similar or different perspectives, as in our study (15,16,17,18,19,20,21,22,23,24,25,26,27,28).

An examination of the literature revealed that DESs decrease recurrent revascularization compared with BMSs (16,29) and show more positive results. In our study, several parameters were used to compare effectiveness, and it was concluded that DES was more effective than BMS. The QALY value of patients treated with DES (0.8924) was found to be higher than that of patients treated with BMS (0.8371). The visual analog scale score of the DES group (72.56) was higher than that (64.03) of the BMS group. Treatment with DES in terms of re-hospitalization (19.23%) is more advantageous than BMS (35%). The mean length of hospital stay (1.59 days) of patients treated with DES was shorter than that (1.93 days) of patients treated with BMS. In studies on this subject, effectiveness was compared with various parameters, and the effectiveness of DES was found to be higher than the effectiveness of BMS (19,23,24,26,27). Some studies in the literature also concluded that DES is more effective in high-risk patients (17,18,20,22).

ICER, calculated with weighted values obtained from the decision tree analysis of cost and effectiveness data, was found to be 28,179.12 TL per additional QALY. From this point of view, DES was found to be very cost effective compared with BMS. In their study, Cohen et al. (15) found DES to be cost effective compared with BMS (2004). In other studies, DES was found to be cost effective in high-risk patients (17,18,20,21). However, Kutluer (28) did not find DES to be cost effective relative to BMS. In this study, it is thought that this result was achieved because the QALY values, which are the criteria of effectiveness, are taken the same for DES and BMS. Bischof et al. (25) and Hill et al. (16) also found that DES was not cost effective compared with BMS. It is thought that different results may occur in different countries, at different times, and in different health systems due to possible changes in the costs of these health technologies.

### Study limitations

The results of the study should be evaluated within the framework of some limitations. The most important of these are the following: due to the inavailability of the weights of the quality of life for Turkey in the EuroQol 5D 5L Health Questionnaire, the life quality weights for Germany were used; because the study is retrospective, each patient had a different duration elapsed after the procedure; there was a lack of contact information for some patients, or missing or incorrect information; were were unable to contact some prospective subjects because they were deceased. Patients who did not respond to all EuroQol 5D 5L Health Questionnaire questions were not included in the study. To be able to make an accurate cost-effectiveness comparison between two health technologies, we did not include patients in whom both BMS and DES were used simultaneously.

In this study, it was determined that DESs are more costly than BMSs, but they are more advantageous in terms of effectiveness. In the cost-effectiveness analysis, it was concluded that DES was very cost effective compared with BMS. In the case of CAD treatment with BMS, the budget effect is 4,728,891,929.42 TL, and the budget effect is 8,022,260,039.81 TL in treatment with DES. From this point of view, it is seen that treatment with DES has a greater effect on the budget.

Considering that human health is more important than book value, using DES in CAD treatment is predicted to create more patient satisfaction. With the amendment made in the National Communiqué on Healthcare Implementation and published in the Official Gazette No. 30175, dated 09/09/2017 (12), the reimbursement for DES was reduced from 1,018.00 TL to 650.00 TL. Considering the effectiveness of DES, it is thought that it would be beneficial for service providers to pay more for this technology. It is recommended that policymakers develop policies to prevent CAD, which has a large budgetary impact.

## Figures and Tables

**Table 1:**
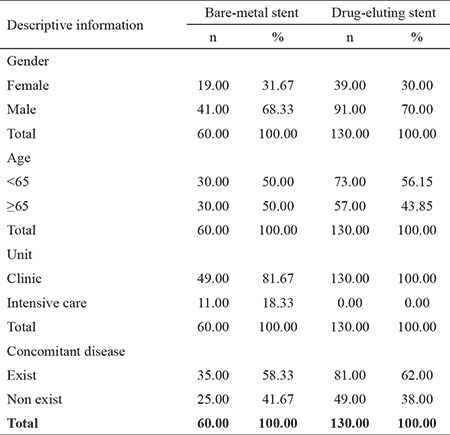
Descriptive findings of the study

**Table 2:**
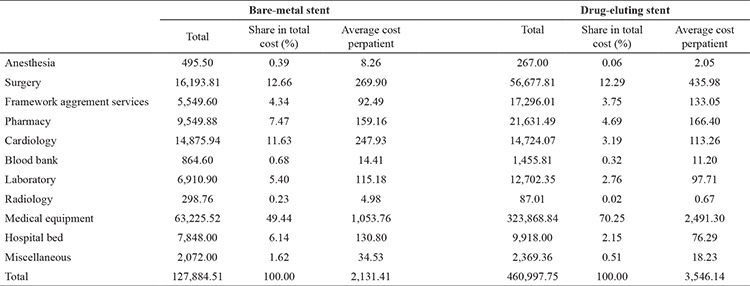
Treatment costs of coronary artery disease (Turkish Liras)

**Table 3:**
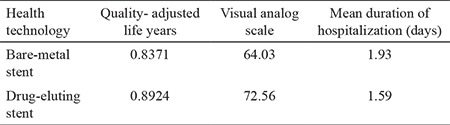
Effectiveness findings

**Table 4:**
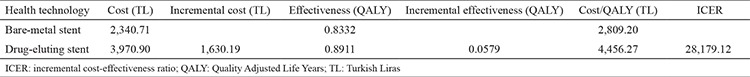
Cost-effectiveness analysis results

**Table 5:**
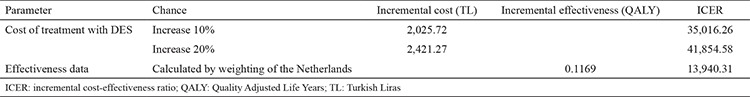
Sensivity analysis findings

**Table 6:**
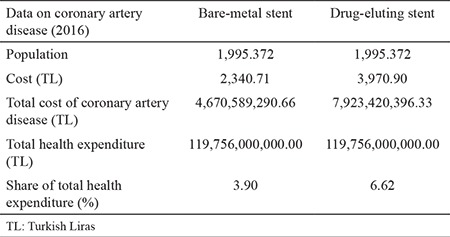
Budget effectiveness analysis findings
